# Editorial: Genetics of Age-Related Diseases and Their Risk and Protective Factors

**DOI:** 10.3389/fgene.2021.771109

**Published:** 2021-09-27

**Authors:** Ida K. Karlsson, Thalida Em Arpawong, Yiqiang Zhan, Kelli Lehto

**Affiliations:** ^1^ Department of Medical Epidemiology and Biostatistics, Karolinska Institutet, Stockholm, Sweden; ^2^ Aging Research Network-Jönköping, School of Health and Welfare, Jönköping University, Jönköping, Sweden; ^3^ Leonard Davis School of Gerontology, University of Southern California, Los Angeles, CA, United States; ^4^ School of Public Health, Sun Yat-Sen University, Guangzhou, China; ^5^ Institute of Genomics, University of Tartu, Tartu, Estonia

**Keywords:** genetic epidemiology, aging, age-related disease, editorial, risk factor, protective factor

Aging is a universal biological process, but with great individual variability, where some remain healthy throughout old age while others suffer from health problems and co-morbidities from a relatively early old age (Melzer et al., 2020). Twin studies have estimated the heritability of the common age-related diseases cardiovascular disease, type 2 diabetes, and Alzheimer’s disease to between 45 and 80% (Zdravkovic et al., 2002; Gatz et al., 2006; Willemsen et al., 2015). This indicates that a substantial amount of the total variance in these conditions is attributable to genetic variation, and that genetic study designs can make important contributions to our understanding of aging processes and age-related diseases. Indeed, genome-wide association studies (GWASs) have identified many genetic variants associated with these conditions (Nelson et al., 2017; Mahajan et al., 2018; Jansen et al., 2019), and other age-related diseases, even if they still explain only part of the estimated heritability. Important insights into disease mechanisms have also been made by using the results from GWASs to understand e.g. biological pathways and disease associations (Melzer et al., 2020). The aim of this research topic was to join high quality research projects in genetics that help us to improve our understanding of age-related diseases as well as the role of their risk and protective factors. In this research topic, six Original Research Articles and one Brief Research Report present important and relevant findings within the fields of genetics and age-related diseases.

Urzi and colleagues investigated genetic influences in sarcopenia, a geriatric disease characterized by loss of muscle mass and strength, and leads to impairment of one’s physical capacity. The genetic influences of sarcopenia are still poorly understood, and the authors here conducted a pilot study testing the single and combined effects of seven selected single nucleotide polymorphisms (SNPs). They identified statistically significant associations between three of the seven SNPs and sarcopenia, namely *MTHFR*, *ACTN3*, and *NRF2*, and show that these three SNPs together explain a large proportion of the variability in the disease.

It is important not only to understand risk and protective factors of a disease, but also what influences the outcomes of the disease. An excellent example is the study by Dungan et al., who conducted a GWAS in a survival analysis setting, examining genetic variants associated with survival among individuals diagnosed with coronary artery disease (CAD). The authors could highlight two SNPs, one of which is novel and one of which reside in the *DAB2IP* gene, also implicated in CAD diagnosis (Harst and Verweij, 2018).

Four studies within the topic focused on dementia and Alzheimer’s disease (AD). Kim et al. used a twin sample to examine the measured (*APOE* genotype) and unmeasured (twin design) genetic effects underlying the relationship between age-related increase in loneliness and dementia risk. The authors showed that although the baseline level, but not change in loneliness, predicted dementia, these associations diminished after adjusting for genetic and environmental confounds.

Three of the articles used a Mendelian randomization (MR) approach to understand causal influences on the risk of dementia. Ware and colleagues used individual-level data from the U.S. Health and Retirement Study in a one-sample MR design, examining causal effects of type-2 diabetes on the risk of cognitive impairment and dementia. While type-2 diabetes was associated with cognitive impairment, but not dementia, the MR analyses indicated that the association is not of a causal nature. This is in agreement with previous two-sample MR studies of type-2 diabetes and AD, based on GWAS summary statistics (Østergaard et al., 2015; Walter et al., 2016; Thomassen et al., 2020), and the study makes an important contribution by demonstrating that the lack of causality is seen also for cognitive impairment, and is robust to adjustment for education and *APOE* ε4 status.

Yu et al. used summary-level data from GWAS to conduct a two-sample MR study demonstrating that shorter telomere length, a marker of biological aging, is causally associated with risk of AD. This strengthens previous findings on the topic (Zhan et al., 2015; Gao et al., 2019; Guo and Yu, 2019), extending the analyses to a larger sample size and using additional methods to test the robustness of the causal association.

In a Brief Research Report, Wu and colleagues examined the association between growth differentiation factor 15 (GDF-15) and neurodegenerative diseases in a two-sample MR study. The authors found GDF-15 to be causally associated with AD, but not with Parkinson’s disease or amyotrophic lateral sclerosis. GDF-15 is involved in inflammation and proposed to be associated with healthy aging and age-related diseases, and the findings presented by Wu et al. indicate that it may be a promising diagnostic marker and therapeutic target for AD.

Genetic research methods are constantly improving, a good example of which is presented in the Research Article by Gao and colleagues. Here, the authors demonstrate that sex-specific two-sample MR studies may be biased if sex heterogeneity in the instrumental variable is not considered, i.e. when the instrumental variable is based on results from men and women combined, while the outcome is sex-specific and based on results from only men or only women. This is demonstrated by examining the causal effects of anthropometric traits on breast and prostate cancer, indicating that instrumental variables based on sex-combined summary data may be biased relative to those based on sex-stratified data.

In summary, the Research Topic “Genetics of Age-Related Diseases and Their Risk and Protective Factors” has brought together articles covering broad and important areas within genetics of age-related diseases. Importantly, the articles demonstrate how genetic methods can be used to advance our understanding of disease mechanisms and provide targets for future hypothesis-driven studies, as well as how to make use of already existing knowledge, data, and findings to better understand how risk and protective factors act to influence etiologies for age-related diseases.
